# Impact of type 2 diabetes mellitus on hematoma expansion and clinical outcomes in patients with spontaneous intracranial hematoma

**DOI:** 10.3389/fsurg.2025.1693924

**Published:** 2025-10-01

**Authors:** Junhua Yang, Sihui Wang, Xiangtian Ji, Yu Sun, Jingyu Feng, Bin Liu, Jun Yang

**Affiliations:** 1 Department of Neurosurgery, Peking University Third Hospital, Beijing, China; 2 Center for Precision Neurosurgery and Oncology, Peking University Health Science Center, Beijing, China

**Keywords:** spontaneous intracranial hematoma, diabetes mellitus, hematoma expansion, prognosis, stroke

## Abstract

**Background and purpose:**

Spontaneous intracerebral hemorrhage (sICH) significantly affects patient outcomes. Diabetes mellitus (DM) is a common comorbidity associated with sICH; however, the influence of DM on hematoma expansion (HE) and clinical prognosis in sICH patients remains a subject of debate.

**Methods:**

This multicenter retrospective study included sICH patients who visited the neurosurgery departments of eight hospitals between 1 January 2015 and 31 May 2021. These patients were followed up for 6 months post-sICH and divided into two groups: those with type 2 DM (DM group) and those with no diabetes mellitus (nDM group). The chi-square test, Mann–Whitney *U* test, and multivariate logistic regression analysis were employed to evaluate the impact of DM on HE and patient outcomes.

**Results:**

A total of 1,453 patients were admitted to the eight hospitals between 1 January 2015 and 31 May 2021. A total of 1,134 sICH patients were ultimately included in this study for further analysis, based on the inclusion and exclusion criteria. Of these, 182 (16.0%) patients were assigned to the DM group, while 952 (84.0%) patients were assigned to the nDM group. In the intergroup comparison, significant differences were observed in terms of gender, age, smoking, hypertension, coronary heart disease, antiplatelet therapy, ventricular hematoma, and heterogeneous hematoma density. After adjusting for the above confounders, DM was found to significantly increase the incidence of HE (3.552, 95% CI: 2.342–5.387, *p* = 0.000). Similarly, DM significantly increased all-cause mortality at 1 month (1.965, 95% CI: 1.006–3.840, *p* = 0.048), 3 months (1.980, 95% CI: 1.071–3.662, *p* = 0.029), and 6 months (1.776, 95% CI: 1.034–3.050, *p* = 0.038) in sICH patients. However, DM did not worsen functional prognosis in patients with sICH at 1 month (1.363, 95% CI: 0.909–2.043, *p* = 0.134), 3 months (1.124, 95% CI: 0.746–1.692, *p* = 0.577), and 6 months (1.177, 95% CI: 0.789–1.754, *p* = 0.425), after adjusting for confounding factors.

**Conclusion:**

DM is a risk factor for HE and all-cause mortality at 1, 3, and 6 months post-sICH. However, DM does not significantly worsen the functional prognosis of sICH patients at 1, 3, and 6 months.

## 1 Introduction

Spontaneous intracranial hemorrhage (sICH) occurs as a result of blood leakage into the brain parenchyma following the rupture of cerebral vessels due to non-traumatic etiologies (1). Although less common than ischemic stroke, sICH is likely the most fatal form of stroke with early-term mortality approximately 30%–40% (2). The development of hematoma expansion (HE) can further aggravate the deterioration of the nervous system and increase the likelihood of adverse clinical outcomes (3).

According to statistical data, approximately 14% of stroke patients are comorbid with diabetes mellitus (DM) (4). This proportion is expected to increase further due to the aging population. Consequently, an increasing number of researchers have started to focus on the impact of DM on the clinical outcomes of sICH patients (5–8). However, the majority of existing clinical studies investigating the impact of DM on sICH involve small sample sizes, and the precise effect remains a subject of debate (9–13).

Therefore, we designed a multicenter retrospective cohort study to further assess the impact of DM on HE and clinical outcomes in patients with sICH.

This study is a multicenter retrospective study. As this study is a retrospective analysis and poses no risk to participants, the Ethics Committee of Peking University Third Hospital waived the requirement for informed consent.

## 2 Materials and methods

### 2.1 Study population

This study is a multicenter retrospective analysis. Based on the predefined inclusion and exclusion criteria, this study consecutively enrolled patients with sICH who presented to the neurosurgery departments of eight medical institutions in Beijing and Guangzhou city between 1 January 2015 and 31 May 2021.

### 2.2 Inclusion and exclusion criteria

#### 2.2.1 Inclusion criteria

(1) Participants must be aged between 18 and 90 years; (2) individuals presenting with cerebral hemorrhage symptoms, including headache, aphasia, and impaired physical activity; (3) sICH confirmed by head computed tomography (CT) within 24 h of symptom onset.

#### 2.2.2 Exclusion criteria

(1) Patients younger than 18 years or older than 90 years; (2) neurological deficits, such as aphasia, or impaired physical activity caused by sICH was not confirmed by brain CT; (3) secondary intracerebral hemorrhage due to aneurysm rupture, cerebral vascular malformation rupture, subarachnoid hemorrhage, amyloidosis-induced cerebral hemorrhage, tumor-related stroke, hemorrhagic transformation of cerebral infarction, or brain trauma; (4) sICH in patients with type 1 DM; (5) patients with malignant tumors or severe dysfunction of the heart, lungs, liver, or kidneys; (6) patients undergoing anticoagulant therapy or with coagulation disorders; (7) patients with incomplete data records.

### 2.3 Treatment

All patients had their baseline CT scan within 24 h of symptom onset and were treated after presentation. The patient's medical team selected either surgical or conservative treatment based on the patient's condition, the family's preferences, and surgical indications. The first follow-up CT scan was routinely performed at 6 h or 24 h after the baseline CT scan or surgery. Then, follow-up CTs were performed every 2–3 days or when patients had new neurological symptoms.

Surgical indications included signs of cerebral herniation, a midline shift exceeding 1 cm, supratentorial intracerebral hemorrhage volume >30 mL, infratentorial bleeding exceeding 10 mL, and progressive worsening of consciousness disturbance (14).

The surgical approaches employed were one or a combination of the following: microscopic evacuation of intracranial hematoma, endoscopic evacuation of intracranial hematoma, and minimally invasive surgery.

### 2.4 Follow-up

Patients with sICH were followed up via telephone after discharge, continuing until 6 months post-sICH. The functional status and survival status of the patients were systematically recorded at 1, 3, and 6 months following the sICH event.

### 2.5 Data collection

Data of sICH patients during hospitalization were retrieved from electronic medical records, while follow-up data for these patients were collected via telephone interviews. The CT scans of all patients were analyzed independently by two double-blind neurosurgeons. The opinion of two physicians was accepted only if it was identical; otherwise, it was reanalyzed by a third physician.

The following clinical and follow-up data were systematically collected: (1) demographic information, including admission number, contact telephone, gender, age, height, and weight; (2) lifestyle factors, such as smoking and alcohol consumption history; (3) medical history, encompassing hypertension, diabetes, coronary heart disease, dyslipidemia, antiplatelet therapy, cerebral infarction history, and cerebral hemorrhage history; (4) radiological characteristics, including hematoma location, side, volume calculated by A × B × C/2 method (15), intraventricular hemorrhage, hematoma density, shape, and specific signs (such as island sign, blend sign, black hole sign); (5) treatment approaches; (6) laboratory findings, such as platelet count, international normalized ratio, and glycemia levels; and (7) Glasgow Coma Scale (GCS), Modified Rankin Scale (mRS) scores and survival status of sICH patients at 1, 3, and 6 months post-sICH.

### 2.6 Outcomes and definition

HE was defined as either a proportional increase in hematoma volume of ≥33% or an absolute increase in volume of ≥6 mL compared with the previous CT findings on any follow-up CT within 1 month after sICH onset (16). mRS score <3 was defined as a good functional outcome at 1, 3, and 6 months post-sICH (17), while mRS score ≥3 was defined as a poor functional outcome at 1, 3, and 6 months post-sICH (17). Mortality was defined as all-cause death at 1, 3, and 6 months post-sICH.

### 2.7 Statistical analysis

All categorical variables in this study were summarized using counts and percentages. Continuous variables were presented as means and interquartile ranges. For comparisons between groups, categorical variables were analyzed using the chi-square test, while continuous variables were assessed using the Mann–Whitney *U* test. Multivariate logistic regression analysis was employed to adjust for confounding factors with a significance level of *p* < 0.05 in between-group comparisons. Statistical analyses, including the chi-square test, Mann–Whitney *U* test, and multivariate logistic regression, were conducted using SPSS 26.0 software (IBM). Bar charts and forest plots were generated using GraphPad Prism 10.0. Bilateral *p* < 0.05 were considered statistically significant.

## 3 Results

### 3.1 Study population

A total of 1,453 patients were continuously diagnosed with sICH in the neurosurgery departments of the above medical centers between 1 January 2015 and 31 May 2021. According to the inclusion and exclusion criteria, 159 patients were excluded due to secondary intracerebral hemorrhage, such as ruptured aneurysm, ruptured cavernous hemangioma, and tumor stroke; 58 patients were excluded due to receiving anticoagulant therapy; 21 patients were excluded due to comorbid type 1 DM; 27 patients were excluded due to comorbid severe heart, lung, and liver or kidney dysfunction; 26 patients were excluded due to brainstem hemorrhage; and 28 patients were excluded due to incomplete data and refusal to sign informed consent. A total of 1,134 sICH patients were finally included in the analysis.

During the follow-up period, 178 patients (15.7%) were lost to follow-up after 3 months. By 6 months, the number of patients lost to follow-up increased to 188, resulting in a loss rate of approximately 16.6%.

### 3.2 Baseline characteristics

The study population was divided into the no diabetes mellitus (nDM) group and the DM group based on whether sICH patients were accompanied by type 2 DM. A total of 952 (84.0%) sICH patients were included in the nDM group, and a total of 182 (16.0%) sICH patients were included in the DM group. The basic characteristics of the two groups are shown in Table 1.

**Table 1:** Patient's baseline characteristics according to DM.

Variables	nDM (*N* = 952, 84.0%)	DM (*N* = 182, 16.0%)	*p*-value
Demographic characteristics			
Gender			
Male	762 (80.0%)	121 (66.5%)	0.000
Female	190 (20.0%)	61 (33.5%)	
Age (years)	55 (45, 63)	62 (56.8, 69)	0.000
Vascular risk factors			
Smoking	370 (38.9%)	51 (28.0%)	0.006
Alcohol	298 (31.3%)	47 (25.8%)	0.141
BMI	25.7 (24.1, 27.8)	25.9 (23.8, 28.0)	0.541
Medical history			
Hypertension	783 (82.2%)	161 (88.5%)	0.040
Coronary heart disease	98 (10.3%)	48 (26.4%)	0.000
Dyslipidemia	89 (9.3%)	23 (12.6%)	0.173
Ischemic stroke history	162 (17.0%)	39 (21.4%)	0.153
Cerebral hemorrhage history	45 (4.7%)	11 (6.045%)	0.452
Antiplatelet therapy	198 (20.8%)	60 (33.0%)	0.000
Imaging			
Side			
Left	473 (49.7%)	88 (48.4%)	0.627
Right	479 (50.3%)	94 (51.6%)	
Localization			
Deep	781 (82.0%)	156 (85.7%)	0.230
Lobar	171 (18.0%)	26 (14.3%)	
Hematoma volume (mL)	43.2 (33.2,61.5)	45.2 (33.8, 62.3)	0.281
Ventricular hematoma	434 (45.6%)	105 (57.7%)	0.003
Island sign	263 (27.6%)	60 (33.0%)	0.144
Blend sign	258 (27.1%)	44 (24.2%)	0.413
Black hole sign	643 (67.5%)	117 (64.3%)	0.392
Heterogeneous hematoma density	589 (61.9%)	130 (71.4%)	0.014
Irregular shape	587 (61.7%)	110 (60.4%)	0.757
Surgery	576 (60.5%)	112 (61.5%)	0.794
Laboratory test			
Platelet count 10^9^ /L			
<125	63 (6.6%)	19 (10.4%)	0.078
125–350	861 (90.4%)	161 (88.5%)	
>350	28 (2.9%)	2 (1.1%)	
International normalized ratio	1.00 (0.95, 1.06)	0.99 (0.95, 1.07)	0.320
Glycemia	7.5 (6.5, 8.7)	12.2 (9.8, 13.5)	0.000
Admission GCS	11 (8, 13)	11 (9, 13)	0.339

DM, diabetes mellitus; nDM, no diabetes mellitus; *N*, number; BMI, body mass index; GCS, Glasgow Coma Scale.

As shown in Table 1, the proportion of males (80.0% vs. 66.5%, *p* = 0.000) and smoking (38.9% vs. 28.0%, *p* = 0.006) in the nDM group was significantly higher than that in the DM group. However, the DM group was older on average than those in the nDM group (62 vs. 55, *p* = 0.000). Additionally, the DM group had a higher proportion of patients with hypertension (88.5% vs. 82.2%, *p* = 0.040), coronary heart disease (26.4% vs. 10.3%, *p* = 0.000), antiplatelet therapy (33.0% vs. 20.8%, *p* = 0.000), ventricular hematoma (57.7% vs. 45.6%, *p* = 0.003), and heterogeneous hematoma density (71.4% vs. 61.9%, *p* = 0.014), as shown in Table 1. Undoubtedly, blood glucose levels were significantly higher in the DM group than those in the nDM group (*p* = 0.000).

In addition, no statistical differences were observed between the two groups of patients in terms of alcohol, body mass index (BMI), dyslipidemia, ischemic stroke history, cerebral hemorrhage history, hematoma location, hematoma volume, hematoma shape, platelet count, international normalized ratio, and admission GCS (*p* > 0.05) as shown in Table 1.

### 3.3 Association between DM and outcomes

Among the 1,134 sICH patients, 277 developed HE, resulting in an overall incidence rate of 24.4%. Specifically, 207 (21.7%) patients in the nDM group developed HE, while 70 (38.5%) patients in the DM group experienced HE. As presented in Table 2, the incidence of HE was significantly higher in the DM group compared with that in the nDM group (*p* = 0.000).

**Table 2:** The association between DM and outcomes.

Outcomes	nDM (*N* = 952, 84.0%)	DM (*N* = 182, 16.0%)	*p*-value
HE	207 (21.7%)	70 (38.5%)	0.000
Prognosis			
One-month mRS			
mRS < 3	323 (33.9%)	46 (25.3%)	0.022
mRS ≥ 3	629 (66.1%)	136 (74.7%)	
One-month mortality	40 (4.2%)	17 (9.3%)	0.004
Three-month mRS			
mRS < 3	377 (46.6%)	49 (33.3%)	0.003
mRS ≥ 3	432 (53.4%)	98 (66.7%)	
Three-month mortality	52 (6.4%)	22 (15.0%)	0.000
Six-month mRS			
mRS < 3	474 (59.2%)	64 (44.1%)	0.001
mRS ≥ 3	327 (40.8%)	81 (55.9%)	
Six-month mortality	72 (9.0%)	31 (21.4%)	0.000

DM, diabetes mellitus; nDM, no diabetes mellitus; *N*, number; HE, hematoma expansion; mRS, modified rankin scale.

**Figure 1:**
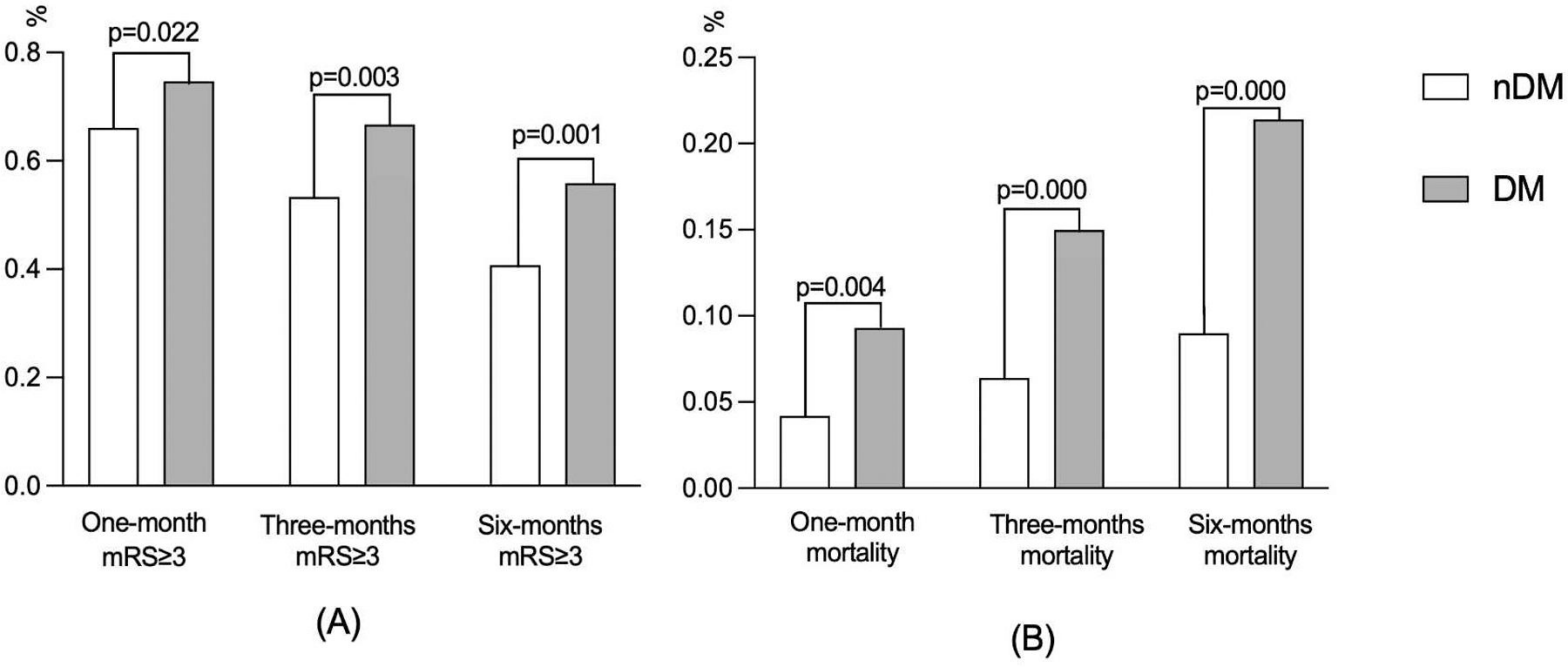
Bar graphs of **(A)** functional prognosis and **(B)** mortality.

During the follow-up, 136 (74.7%) patients, 98 (66.7%) patients, and 81 (55.9%) patients in the DM group had a poor prognosis at 1, 3, and 6 months, respectively. Meanwhile, there were 629 (66.1%), 432 (53.4%), and 327 (40.8%) patients with poor prognosis in the nDM group, respectively. As shown in Table 2 and Figure 1A, the incidence of poor prognosis in the DM group was significantly higher than that in the nDM group at 1 month (*p* = 0.022), 3 months (*p* = 0.003), and 6 months (*p* = 0.001). Furthermore, as shown in Figure 1A, the rate of poor prognosis in both groups demonstrated a downward trend over time.

Similarly, there were 17 (9.3%), 22 (15.0%), and 31 (21.4%) deaths in the DM group at 1, 3, and 6 months. Meanwhile, there were 40 (4.2%), 52 (6.4%), and 72 (9.0%) deaths in the nDM group, respectively. As shown in Table 2 and Figure 1B, all-cause mortality was significantly higher in the DM group than in the nDM group at 1 month (*p* = 0.004), 3 months (*p* = 0.000), and 6 months (*p* = 0.000). Furthermore, the mortality of patients in both groups showed an increasing trend over time, as shown in Figure 1B.

### 3.4 Effect of DM on HE

To account for potential confounding factors between groups, variables with *p* < 0.05 in the intergroup comparison were incorporated into the multivariate analysis for adjustment. After adjusting for gender, age, smoking, hypertension, coronary heart disease, antiplatelet therapy, ventricular hematoma, and heterogeneous hematoma density, DM (3.552, 95% CI: 2.342–5.387, *p* = 0.000) was found to significantly increase the incidence of HE, as shown in Table 3. Additionally, the researchers identified female and heterogeneous hematoma density as risk factors for HE.

**Table 3:** The effect of DM on HE after adjustment.

Variables	HE	
	Odds ratio (95% CI)	*p*-value
DM	3.552 (2.342–5.387)	0.000
Gender	0.072 (0.027–0.189)	0.000
Age	0.988 (0.976–1.001)	0.071
Smoking	0.744 (0.549–1.007)	0.055
Hypertension	0.823 (0.526–1.288)	0.395
Coronary heart disease	0.830 (0.498–1.382)	0.474
Antiplatelet therapy	1.616 (0.876–2.982)	0.124
Ventricular hematoma	1.322 (0.979–1.785)	0.068
Heterogeneous hematoma density	3.404 (2.165–5.352)	0.000

DM, diabetes mellitus; HE, hematoma expansion; CI, confidence interval.

### 3.5 Effect of DM on functional prognosis

**Figure 2:**
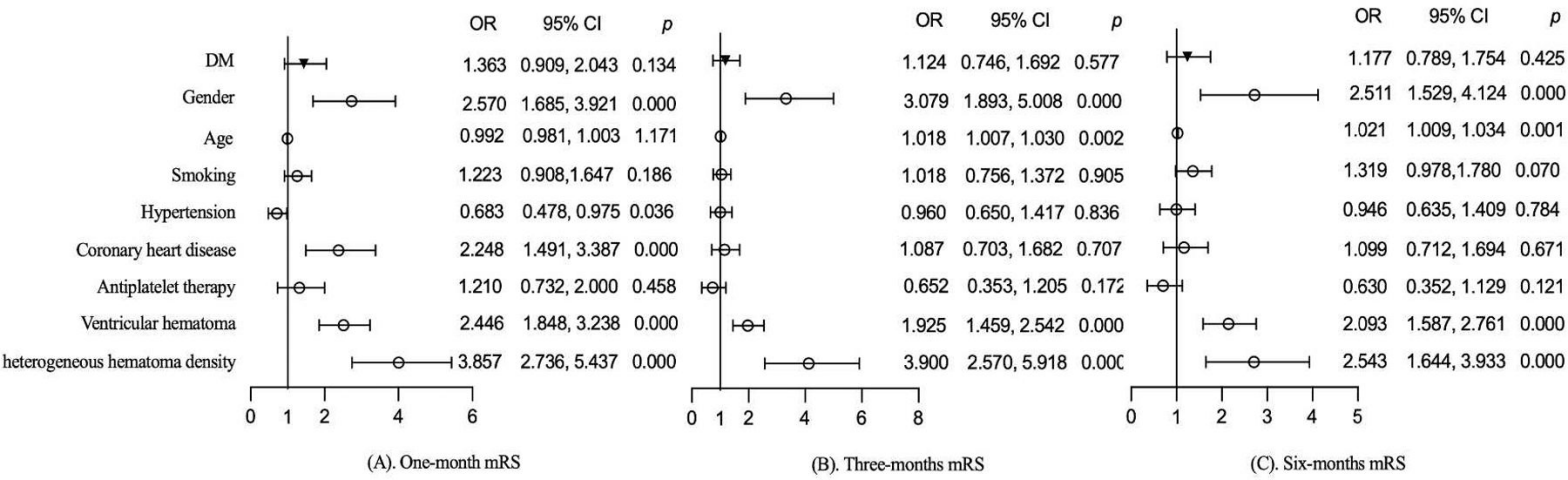
Effect of DM on **(A)** 1-month mRS, **(B)** 3-month mRS, and **(C)** 6-month mRS.

The effects of DM on functional prognosis at different follow-up periods were further evaluated in the follow-up population. Variables with *p* < 0.05 for comparison between groups were included in the multivariate analysis for adjustment. After adjusting for gender, age, smoking, hypertension, coronary heart disease, antiplatelet therapy, ventricular hematoma, and heterogeneous hematoma density, DM did not worsen functional prognosis in patients with sICH at 1 month (1.363, 95% CI: 0.909–2.043, *p* = 0.134), 3 months (1.124, 95% CI: 0.746–1.692, *p* = 0.577), and 6 months (1.177, 95% CI: 0.789–1.754, *p* = 0.425), as shown in Figure 2. Additionally, gender, ventricular hematoma, and heterogeneous hematoma density were also identified as risk factors for functional prognosis, as shown in Figure 2.

### 3.6 Effect of DM on survival prognosis

**Figure 3:**
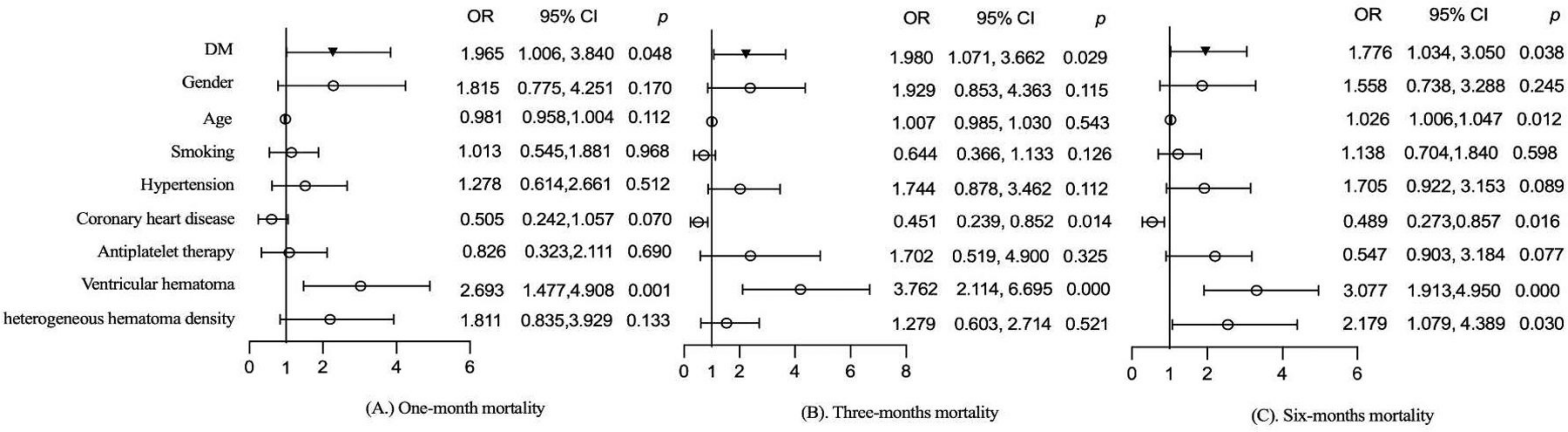
Effect of DM on **(A)** 1-month mortality, **(B)** 3-month mortality, and **(C)** 6-month mortality.

The effects of DM on mortality at different follow-up periods were also evaluated. After adjusting for gender, age, smoking, hypertension, coronary heart disease, antiplatelet therapy, ventricular hematoma, and heterogeneous hematoma density, DM significantly increased all-cause mortality at 1 month (1.965, 95% CI: 1.006–3.840, *p* = 0.048), 3 months (1.980, 95% CI: 1.071–3.662, *p* = 0.029), and 6 months (1.776, 95% CI: 1.034–3.050, *p* = 0.038) in sICH patients, as shown in Figure 3. Additionally, ventricular hematoma was identified as a risk factor for mortality, as shown in Figure 3.

## 4 Discussion

In this multicenter retrospective cohort study, which included 1,134 patients with sICH from the eight clinical practices, patients with DM were found to be at an increased risk of developing HE following sICH and exhibited higher all-cause mortality rates at 1, 3, and 6 months post-sICH. However, no significant differences were observed in the 1, 3, and 6 months functional prognosis between patients with DM and those without DM.

According to the report, the incidence of HE is approximately 30% in patients with sICH (18). This is consistent with the present study, in which the incidence of HE was 24.4% in the population, and DM was found to be a risk factor for HE in sICH patients. However, some clinical investigators have observed contrary results (12, 19). Zhang et al. (12) and Takeda et al. (19) did not identify a significant difference in DM prevalence between HE patients and non-HE patients when comparing the two groups. However, the study population involved in their study was relatively small, especially the limited number of HE cases. This may have limited the accuracy of their findings. In order to accurately evaluate the effect of DM on HE in patients with sICH, many researchers have done many studies and developed predictive models that can accurately predict the occurrence of HE. In 2019, Du et al. (20) analyzed the clinical data of 402 patients and developed a logistic regression model for HE. They revealed that DM significantly increased the incidence of HE in sICH patients within this model (20). Consistent findings were reported in a subsequent study by Yang et al. (21), which indicated that the odds of HE were 1.972 times higher in patients with DM compared with those without DM. In addition, Zhu et al. (13) also identified that hyperglycemia was associated with an increased risk of HE during their investigation into the relationship between systemic inflammatory response syndrome and HE. These studies are consistent with the results of our study; however, we did not further stratify the blood glucose level of sICH in this study. The level of blood glucose in DM patients may have an impact on the occurrence of HE in sICH patients. In 2023, Zhou et al. (22) calculated the average, maximum, minimum, and standard deviation and coefficient of variation of blood glucose levels for each sICH patient in their study and conducted correlation analysis with the clinical prognosis of the patient. As reported, maximal blood glucose level (OR, 1.256; 95% CI, 1.124–1.404; *p* < 0.001) was found to be an independent predictor of HE in sICH patients (22). A prospective cohort study in 2022 also found that the stress-induced hyperglycemia group had a higher lung infection rate and 90-day mortality compared with the non-diabetic euglycemia group (23). Recently, Lu et al. (24) also reported that mean glucose levels, mean amplitude of glycemic excursions, and large amplitude of glycemic excursions were significant predictors of neurological deterioration in sICH patients. This seems to suggest that blood glucose levels can influence the prognosis of patients with sICH. Preclinical studies in mice demonstrated that this outcome may be associated with the compromise of the blood–brain barrier (BBB) (25). This phenomenon has also been observed in human-related studies (26–28). For example, increased BBB permeability with magnetic resonance imaging was detected in patients with DM than in the nDM patients, particularly in the basal ganglia (29). The basal ganglia region is also a common target for sICH. Therefore, it is necessary to strictly control blood glucose in sICH patients with DM.

In addition, researchers have also identified gender, hypertension, CT imaging signs, and white matter lesions as potential risk factors for HE (30–34). In 2017, Marini et al. (32) conducted a comparative analysis of differences in sICH severity, expansion, and mortality between genders. The findings indicated that male sex (1.7, 95% CI: 1.15–2.50, *p* = 0.007) was an independent risk factor for HE (32). A recent meta-analysis provides further evidence supporting the hypothesis that males are at a higher risk of developing HE compared with females (34). However, in this study, the researchers observed distinct outcomes. Specifically, our findings indicate that females may have a higher risk of developing HE compared with males. This could potentially be attributed to the comparatively elevated vascular risk among DM female patients. It has been reported that women with DM exhibit a 27% higher relative risk of stroke compared with men, both for fatal and non-fatal events (35). In addition, this study identified heterogeneous hematoma density as a significant risk factor for HE, which is consistent with the findings reported in prior studies (36–39). In addition, other researchers have observed that antiplatelet therapy, atherosclerosis, ventricular hematoma, and magnesium ions may serve as potential risk factors for HE (40–43). However, no statistically significant difference was observed in the present study.

Except for HE, researchers have also demonstrated a significant interest in the impact of DM on the clinical prognosis of sICH patients. In 2024, Tseng et al. (6) indicated that while DM is significantly associated with poor long-term functional outcomes in patients with sICH, it does not appear to have a significant impact on functional outcomes at 3 and 6 months post-sICH. This finding is in alignment with the results of our study, which indicates that DM did not have a significant impact on the functional outcomes of sICH patients at 1, 3, and 6 months post-sICH. Similar findings were also observed in a study examining motor function outcomes (44). However, some other studies have suggested different conclusions. In 2023, Lusk et al. (45) conducted a secondary analysis of the Ethnic and Racial Variations in Intracerebral Hemorrhage (ERICH) study population. The results indicated that DM was associated with poor functional outcomes at 6 months post-sICH after adjusting for confounding factors (45). Similarly, Pasi et al. (46) reported a comparable conclusion in their study evaluating the impact of DM on long-term functional outcomes in sICH patients, indicating that DM is associated with functional decline over an average follow-up period of 9 years. These were different from the results of our study. In the univariate analysis of our study, functional prognosis differed significantly between the two groups. However, there was no significant difference in multivariate analysis. This may be related to the different study population ethnicity and basal blood glucose levels. As reported, Hispanic patients had higher odds of unfavorable outcomes when they had a history of diabetes compared with non-Hispanic White and non-Hispanic Black patients (45). Further investigations of blood glucose levels also seem to confirm the difference in prognosis caused by different basal blood glucose levels. As reported, hyperglycemia has been documented to be inversely correlated with functional outcomes in nDM patients with sICH (7, 47). This may be attributable to stress hyperglycemia (8, 48). Previous studies have reported an association between stress hyperglycemia and poorer mRS scores (17). In addition, the rehabilitation treatment received by the patient and complications caused by DM may also be factors affecting the functional outcome of sICH patients. Therefore, a substantial amount of prospective data is required to accurately assess the impact of DM on the functional outcomes of sICH patients, thereby excluding potential biases.

In this study, the researchers also examined the mortality rates among different patient groups. The findings revealed that DM was significantly associated with increased all-cause mortality at 1, 3, and 6 months post-sICH. This is consistent with the results of previous studies (9, 11, 49, 50). In 2016, Boulanger et al. (9) assessed the impact of DM on short-term mortality among patients with sICH. Their findings indicated that the 30-day or in-hospital mortality rate was 1.52 times higher in patients with DM compared with those without DM (9). Subsequently, Liebkind et al. (49), evaluating the impact of DM on the long-term mortality of sICH patients in their study, also demonstrated that DM substantially elevates the 1-year mortality risk among sICH patients. The study conducted by Forti et al. (11) even demonstrated that pre-DM was also associated with increased mortality among patients with sICH. However, the study by Boulanger et al. (51) drew a contrasting conclusion. While there was a slight increase in case mortality among patients with type 2 DM in this study, the difference was not statistically significant (51). Similarly, Chen et al. (23) also reported that DM did not significantly affect the 1-month and 3-month mortality rates of sICH patients when blood glucose levels and DM history were categorized and analyzed in their study. This may be attributable to the limited size of the study population and the disparities between the groups. Further high-level clinical evidence is required to validate the effect of DM on sICH mortality.

As a multicenter cohort study, this research is capable of mitigating population bias to a certain extent, thereby enhancing the generalizability of the conclusions. In this study, the enrolled participants were followed up for 6 months via telephone, facilitating a comprehensive evaluation of the prognosis in sICH patients. Additionally, we conducted an assessment of HE, functional outcomes, and survival prognoses, providing valuable insights for clinical practice. Nevertheless, this study has several limitations that warrant acknowledgment. First, constrained by the original data records, we were unable to stratify blood glucose levels within the study population, which may introduce potential biases into the results. Furthermore, we were unable to differentiate between stress hyperglycemia and chronic hyperglycemia. The former may reflect the severity of the sICH insult and is independently associated with outcomes. Therefore, more refined metrics in our future studies will be included, such as glycemic variability, glycosylated hemoglobin (HbA1c), or stress-induced hyperglycemia ratio, to further elucidate the causal relationship between hyperglycemia and prognosis. Third, the study population had a high rate of loss to follow-up, which introduced selection bias. This may have weakened the efficacy of DM for the outcomes of sICH patients. Fourth, the retrospective nature of this study has inherent selection bias, which can reduce the accuracy of the findings. In addition, we only counted the functional and survival status of patients who were discharged from the hospital, and we did not record and analyze the subsequent treatment of patients. This may have made us miss some important information. Therefore, in future work, the research team hopes to conduct a more detailed prospective study to further explore the exact impact of DM on sICH and the best glycemic management strategy.

## 5 Conclusions

DM is a risk factor for HE and all-cause mortality at 1, 3, and 6 months post-sICH. However, DM does not significantly worsen the functional prognosis of sICH patients at 1, 3, and 6 months. Aggressive glucose control therapy may be warranted in patients with sICH.

## Data Availability

The original contributions presented in the study are included in the article/Supplementary Material; further inquiries can be directed to the corresponding author.
